# Supplementary material for: Perinatal development of structural thalamocortical connectivity
Source: Imaging Neurosci (Camb). 2025 Jan 8;3:imag_a_00418. doi: 10.1162/imag_a_00418 (PMC12319864; doi:10.1162/imag_a_00418)
Supplement: Supplementary Material [file imag_a_00418-supp.pdf]

Supplementary material

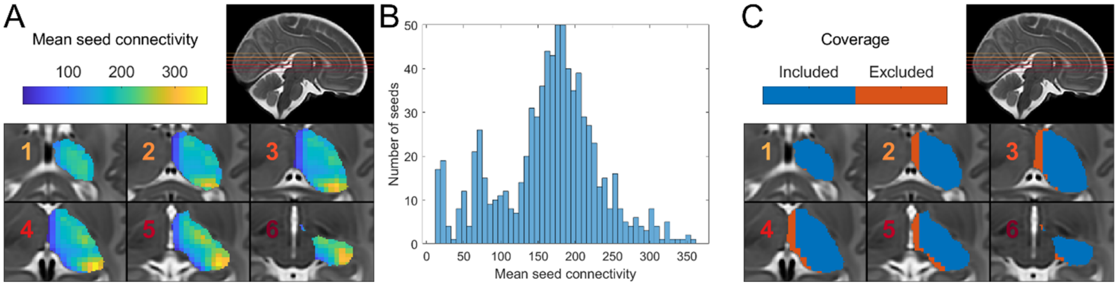

Figure S1. Distribution of non-normalised connectivity values for each seed. (A) The non-normalised connectivity from each thalamic seed to the cortex (averaged across all neonates). (**B**) Histogram of seed connectivity values. (**C**) Final selected seed coverage of the thalamus. Orange areas, whilst covered by the thalamic mask, were excluded as they showed noticeably weaker connectivity than other thalamic areas.

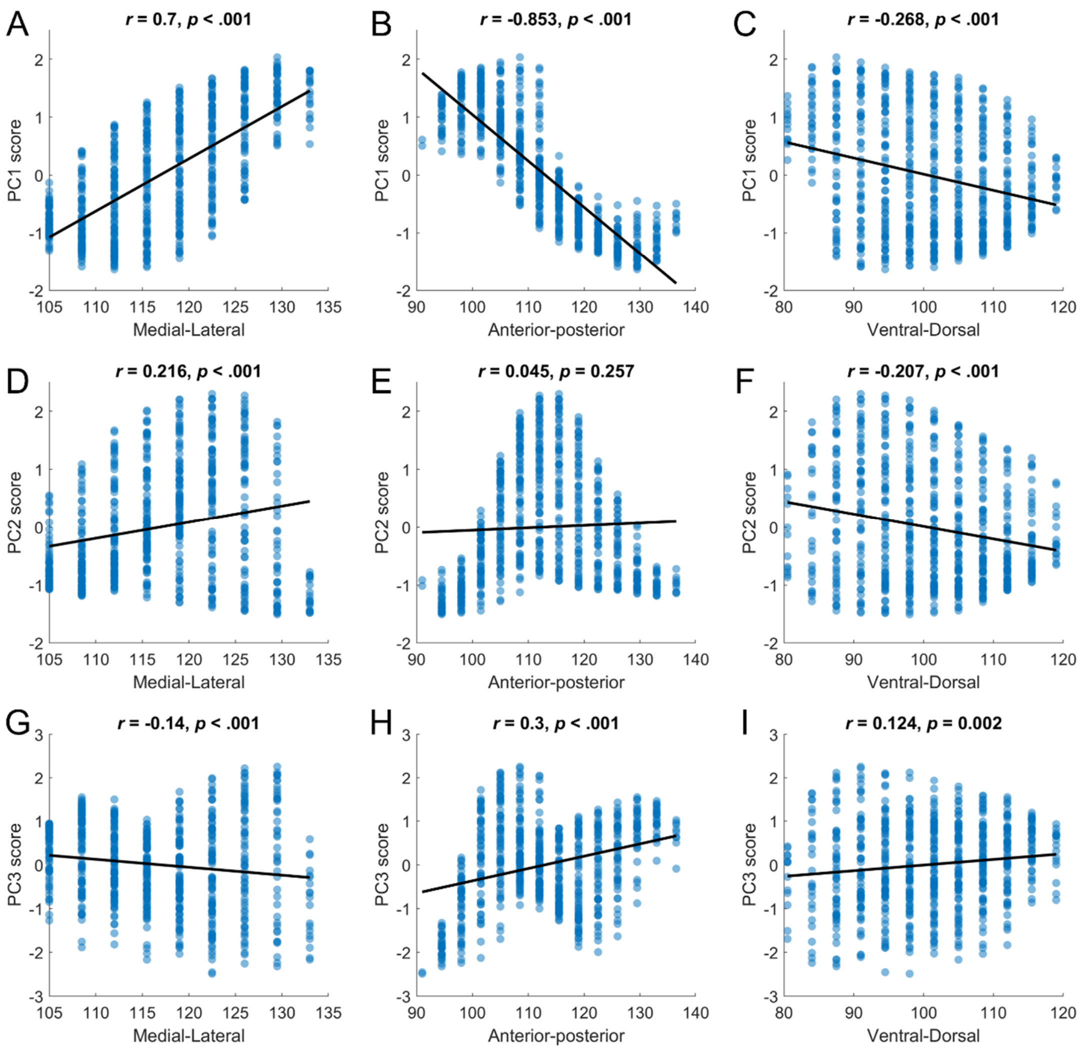

Figure S2. Correlations between seed PC1-PC3 score and cartesian axes position. (A) PC1 score with Medial-lateral axis (*x*-axis voxel coordinate). **(B)** PC1 score with anterior-posterior axis (*y*-axis voxel coordinate). **(C)** PC1 score with dorsal-ventral axis (*z*-axis voxel coordinate). **(D)** PC2 score with Medial-lateral axis (*x*-axis voxel coordinate). **(E)** PC2 score with anterior-posterior axis (*y*-axis voxel coordinate). **(F)** PC2 score with dorsal-ventral axis (*z*-axis voxel coordinate). **(G)** PC3 score with Medial-lateral axis (*x*-axis voxel coordinate). **(H)** PC3 score with anterior-posterior axis (*y*-axis voxel coordinate). **(I)** PC3 score with dorsal-ventral axis (*z*-axis voxel coordinate). The black line indicates the (linear) line of best fit.

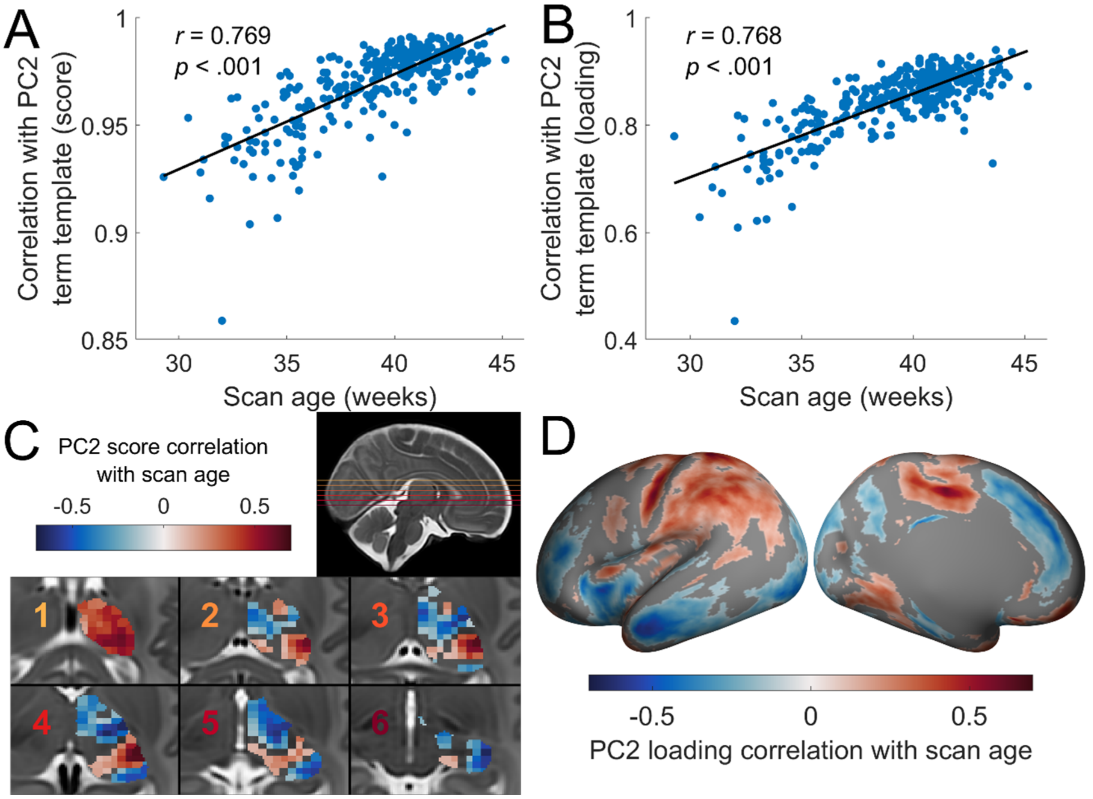

Figure S3. Age-related changes in the secondary thalamocortical axis (**A**) Scatter plot of the correlation between individual and template PC2 scores and individual scan age. (**B**) Scatter plot of the correlation between individual and template PC2 loading and individual scan age. (**C**) Correlation between individual PC2 scores and scan age for each thalamic seed (voxels are coloured according to the value of the nearest seed; non-significant areas are not coloured; $p_{FDR} < 0.05$). (**D**) Correlation between individual PC2 loading and scan age for each cortical vertex (non-significant areas are not coloured; $p_{FDR} < 0.05$).

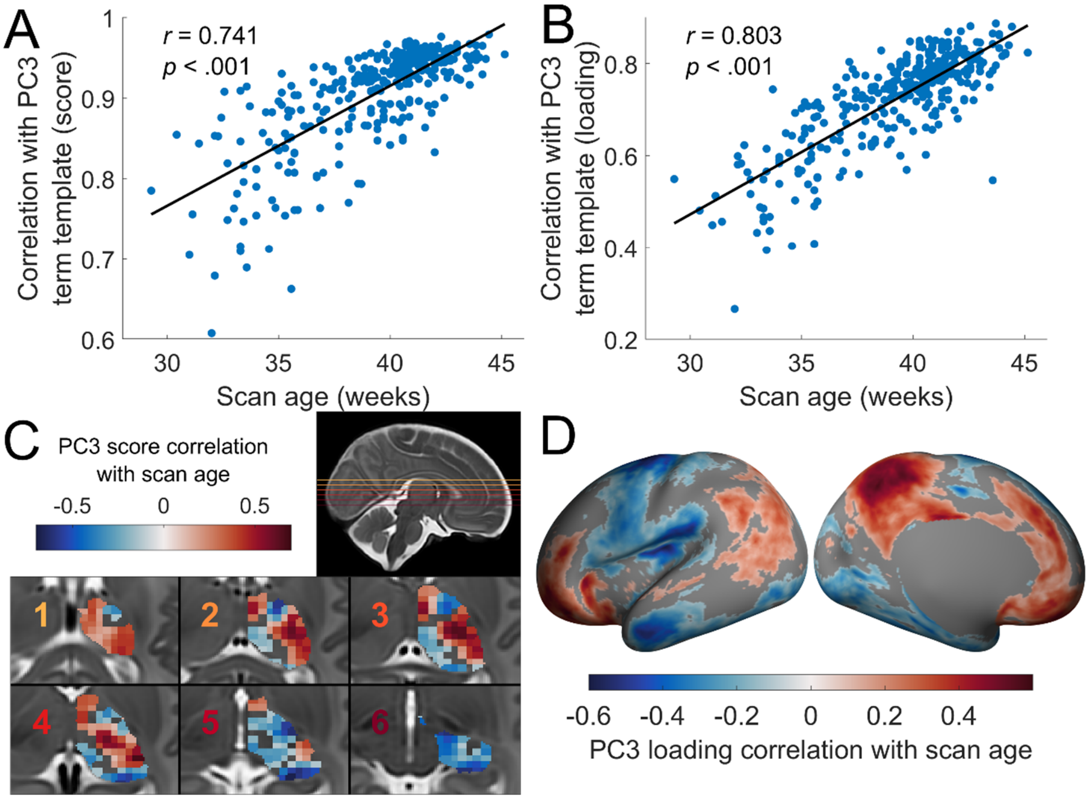

Figure S4. Age-related changes in the tertiary thalamocortical axis (**A**) Scatter plot of the correlation between individual and template PC3 scores and individual scan age. (**B**) Scatter plot of the correlation between individual and template PC3 loading and individual scan age. (**C**) Correlation between individual PC3 scores and scan age for each thalamic seed (voxels are coloured according to the value of the nearest seed; non-significant areas are not coloured; $p_{FDR} < 0.05$). (**D**) Correlation between individual PC3 loading and scan age for each cortical vertex (non-significant areas are not coloured; $p_{FDR} < 0.05$).

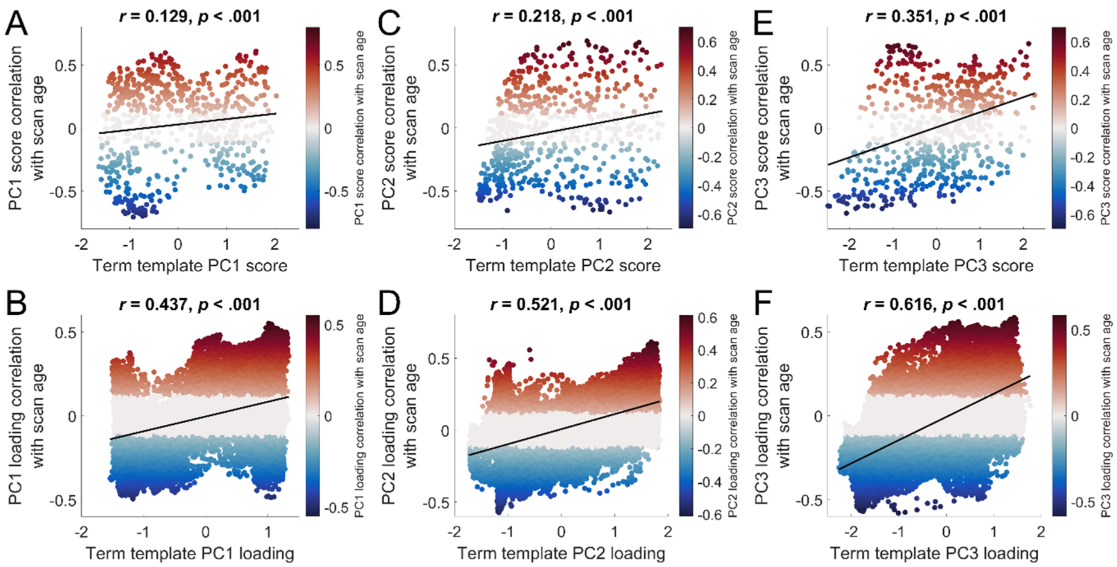

Figure S5. Relationship between age-related gradient changes and gradient position. (**A**) Relationship between the term template PC1 score and PC1 score age-related changes. (**B**) Relationship between the term template PC1 loading and PC1 loading age-related changes. (**C**) Relationship between the term template PC2 score and PC2 score age-related changes. (**D**) Relationship between the term template PC2 loading and PC2 loading age-related changes. (**E**) Relationship between the term template PC3 score and PC3 score age-related changes. (**F**) Relationship between the term template PC3 loading and PC3 loading age-related changes. The black line indicates the line of best fit.

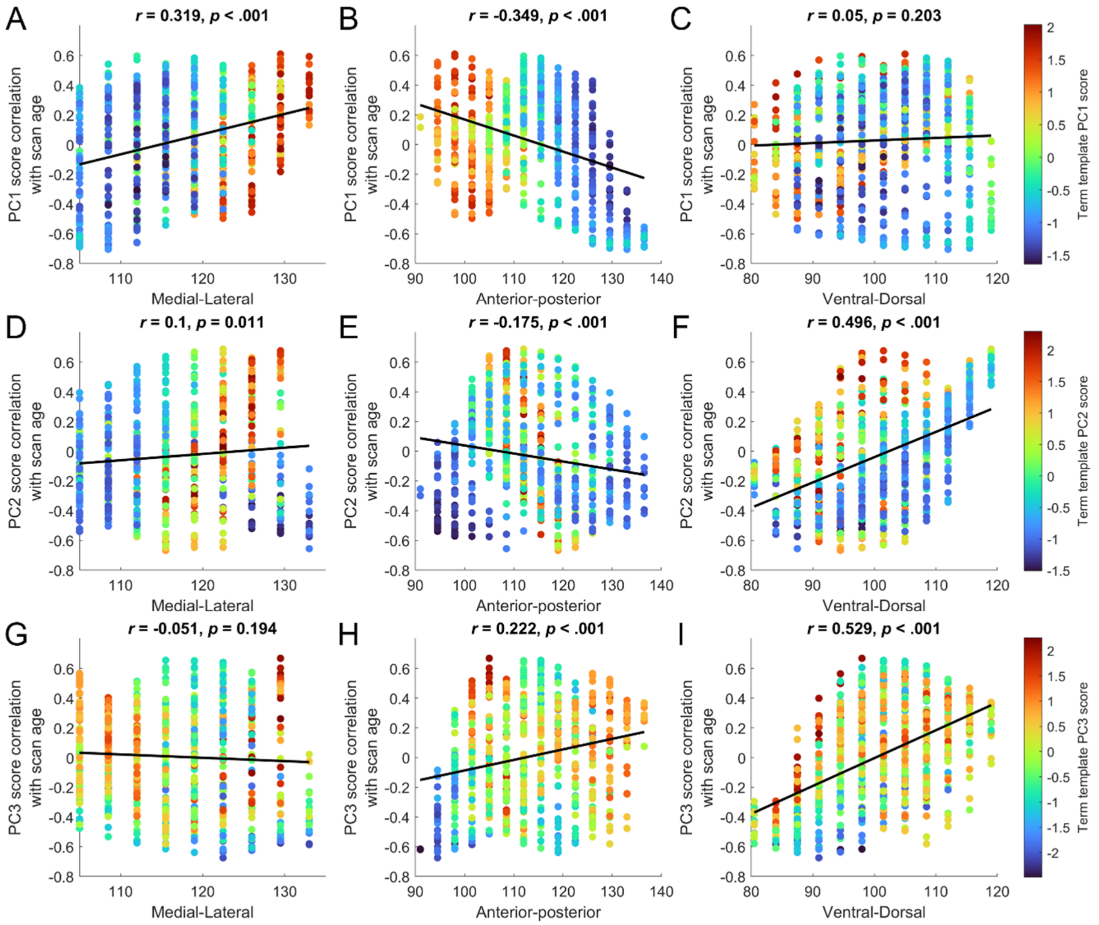

Figure S6. Correlations between age-related changes in PC score and cartesian axes position. **(A)** Relationship between age-related changes in PC1 scores and with medial-lateral axis position (*x*-axis voxel coordinate). **(B)** Relationship between age-related changes in PC1 scores and with anterior-posterior axis (*y*-axis voxel coordinate). **(C)** Relationship between age-related changes in PC1 scores and with dorsal-ventral axis (*z*-axis voxel coordinate). **(D)** Relationship between age-related changes in PC2 scores and with medial-lateral axis position (*x*-axis voxel coordinate). **(E)** Relationship between age-related changes in PC2 scores and with (*y*-axis voxel coordinate). **(F)** Relationship between age-related changes in PC2 scores and with (*z*-axis voxel coordinate). **(G)** Relationship between age-related changes in PC3 scores and with medial-lateral axis position (*x*-axis voxel coordinate). **(H)** Relationship between age-related changes in PC3 scores and with anterior-posterior axis (*y*-axis voxel coordinate). **(I)** Relationship between age-related changes in PC3 scores and with dorsal-ventral axis (*z*-axis voxel coordinate). The black line indicates the line of best fit.

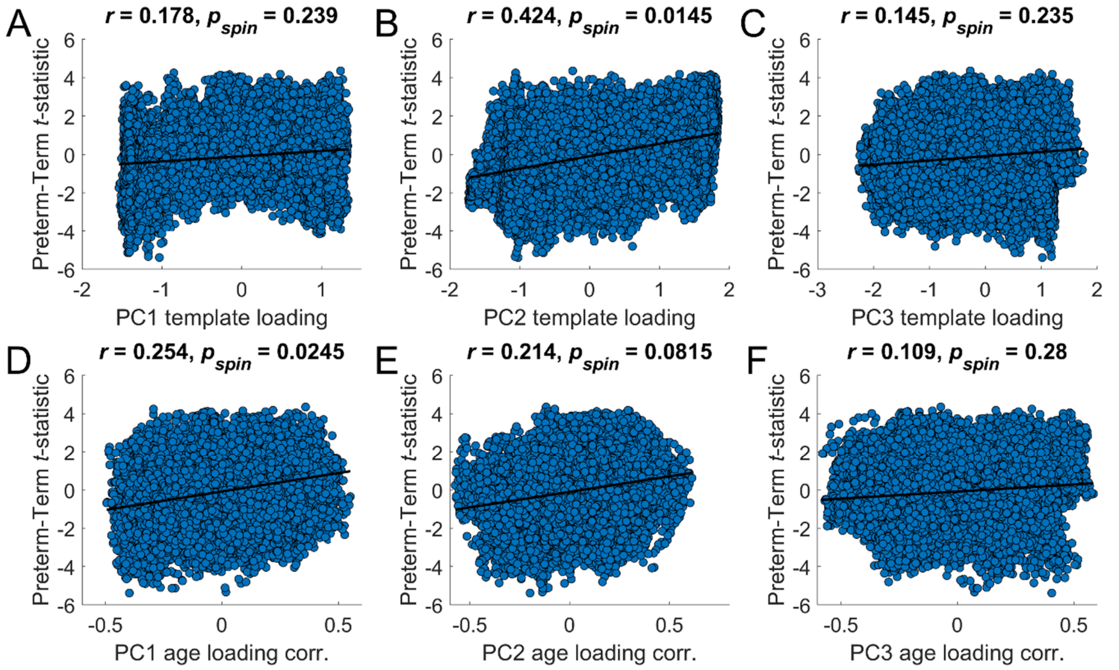

Figure S7. Relationship between cortical thalamocortical connectivity preterm-term differences and gradient position/age-related changes. (**A**) Relationship between preterm-term thalamic connectivity difference and PC1 loading. (**B**) Relationship between preterm-term thalamic connectivity difference and PC2 loading. (**C**) Relationship between preterm-term thalamic connectivity difference and PC3 loading. (**D**) Relationship between preterm-term thalamic connectivity difference and PC1 loading age-related changes. (**E**) Relationship between preterm-term thalamic connectivity difference and PC2 loading age-related changes. (**F**) Relationship between preterm-term thalamic connectivity difference and PC3 loading age-related changes. The black line indicates the line of best fit. Significance was determined using a spin test ($p_{spin} < .05$).
